# Interplay of Inflammation, Immunity, and Organ-Specific Adiposity with Cardiovascular Risk

**DOI:** 10.1155/2014/340847

**Published:** 2014-09-14

**Authors:** Massimiliano M. Corsi Romanelli, Gianluca Iacobellis, Massimo Locati

**Affiliations:** ^1^Chair of Clinical Pathology, Department of Biomedical Sciences for Health, University of Milan, Via Luigi Mangiagalli 31, 20133 Milan, Italy; ^2^Operative Unit of Laboratory Medicine, IRCCS Policlinico San Donato, Milan, Italy; ^3^Chair of Endocrinology, University of Miami, Miller School of Medicine, Miami, FL, USA; ^4^Chair of General Pathology, Department of Translational Medicine, University of Milan, Italy; ^5^Laboratory of Leukocyte Biology, IRCCS Istituto Clinico Humanitas, Rozzano, Milan, Italy

Adipose tissue is a metabolically active organ with anatomical and functional contiguity to many different organs and also myocardium. Under physiological conditions, adipose tissue displays biochemical properties; under pathological circumstances, adipose tissue can affect the heart and vessels through vasocrine and/or paracrine secretion of proinflammatory molecules, such as cytokines, chemokines, and adipokines [1].

Obesity can be considered a state of chronic, low-grade inflammation [2]; particularly visceral adipose tissue (VAT) seems to be an active compartment in proinflammatory molecule secretion [3].

In this special issue, five papers are devoted to explain the linkage between inflammation and organ specific adiposity correlated with cardiovascular risk.

The paper of D. Lio et al. focused attention on a Toll Like receptor, the TLR-4, and its importance in immunity and cardiovascular risk. Moreover, environmental factors play an important role in the interplay of inflammation, organ adiposity, and cardiovascular risk. In fact, M. Greco et al. propose the Mediterranean diet as a scavenger for it.

E. De Falco et al. proposed that adiponectin plays an important role in anticoagulated patients.

M. Bova et al. describe an association between TGF-*β* and thoracic aortic aneurysm. Last but not least, a careful analysis of correlation among carotid stenosis and RANKL is presented by S. Lenglet et al.

We hope that readers will find in this special issue not only accurate data and update review on the mechanisms of interplay of immunity, organ adiposity, and cardiovascular risk, but also important questions to be resolved such as innate immunity role [4], prevention, the effect of health care system, and the role of new molecules.

*Massimiliano M. Corsi Romanelli* *Massimiliano M. Corsi Romanelli* *Gianluca Iacobellis* *Gianluca Iacobellis* *Massimo Locati* *Massimo Locati*

## References

[1] Iacobellis G, Malavazos AE, Corsi MM (2011). Epicardial fat: from the biomolecular aspects to the clinical practice. *The International Journal of Biochemistry & Cell Biology*.

[2] Malavazos AE, Corsi MM, Ermetici F (2007). Proinflammatory cytokines and cardiac abnormalities in uncomplicated obesity: relationship with abdominal fat deposition. *Nutrition, Metabolism and Cardiovascular Diseases*.

[3] Malavazos AE, Cereda E, Morricone L, Coman C, Corsi MM, Ambrosi B (2005). Monocyte chemoattractant protein 1: a possible link between visceral adipose tissue-associated inflammation and subclinical echocardiographic abnormalities in uncomplicated obesity. *European Journal of Endocrinology*.

[4] Locati M, Mantovani A, Sica A (2013). Macrophage activation and polarization a san adaptive component of innate immunity. *Advances in Immunology*.

